# De Novo Heterozygous *GATA3* Missense Variant Causes an Unexpected Phenotype of Non-Syndromic Hearing Impairment with Apparently Recessive Inheritance

**DOI:** 10.3390/ijms26136363

**Published:** 2025-07-02

**Authors:** María Domínguez-Ruiz, Gema Garrido, Paz Martínez-Beneyto, Francisco J. del Castillo, Manuela Villamar, Elena Gómez-Rosas, Miguel A. Moreno-Pelayo, Ignacio del Castillo

**Affiliations:** 1 Servicio de Genética, Hospital Universitario Ramón y Cajal, Instituto Ramón y Cajal de Investigación Sanitaria (IRYCIS), 28034 Madrid, Spain; 2 Centro de Investigación Biomédica en Red de Enfermedades Raras (CIBERER), 28034 Madrid, Spain; 3 ENT Department, Hospital Clínico Universitario, Instituto de Investigación Sanitaria del Hospital Clínico de Valencia (INCLIVA), 46010 Valencia, Spain; 4 Surgery Department, Universitat de Valencia, 46010 Valencia, Spain

**Keywords:** non-syndromic hearing impairment, HDR syndrome, *GATA3*, de novo genetic variant, germline mosaicism

## Abstract

Hearing impairments (HIs) are clinically and genetically very heterogeneous. Finding the causative mutations in patients is frequently a challenge. We investigated two brothers affected by a sensorineural, moderate non-syndromic HI. Exome sequencing revealed that they carried the heterozygous c.812C>T (p.Ser271Leu) variant in *GATA3*. This gene encodes a transcription factor involved in embryonic development, its mutations causing the autosomal dominant HDR (hypoparathyroidism, deafness, and renal disease) syndrome. The variant affects a conserved residue within the proximal zinc-finger motif of GATA3. Sanger sequencing confirmed the presence of the variant in the two brothers, but it showed that surprisingly it was not carried by any of the parents. Segregation studies on 20 fully informative microsatellite markers in the family confirmed that the variant arose de novo. A benign SNP in the mother, close to the position of the variant, allowed us to determine that this was inherited from the father. Gene reporter functional assays supported the pathogenicity of the variant. Clinical reassessment of the two brothers did not disclose any additional abnormality. We conclude that mosaicism for this de novo mutation in the father’s germ line explains the pattern of inheritance in this family and that p.Ser271Leu is causing this unexpected phenotype of non-syndromic HI.

## 1. Introduction

Hearing impairment (HI) is found in a vast number of disorders, which are highly heterogeneous in their etiology and clinical manifestations [1]. HI can be the only clinical sign (isolated or non-syndromic HI), or it can be associated with clinical signs in organs other than the ear (syndromic HI). Over 400 syndromes that include HI have been described, and so the organs involved in these associations of clinical signs are very diverse. A majority of cases have a genetic etiology, with several hundreds of genes involved in syndromic HI and over 150 genes involved in non-syndromic HI [1,2]. These genes encode proteins (and some RNAs) that are essential for the normal development and/or normal function of the ear [3,4].

This huge heterogeneity poses a challenge to genetic diagnosis, given the large number of genes that are involved and given that the syndromic/non-syndromic boundary is rather diffuse. In many forms of syndromic HI, clinical signs in organs other than the ear can manifest much later than the HI, leading to an initial diagnosis of non-syndromic HI. Variable expressivity may also be misleading. Moreover, different mutations in the same gene can result in syndromic or non-syndromic HI [5].

Barakat syndrome, also known as HDR syndrome (for hypoparathyroidism, sensorineural deafness, and renal dysplasia) (OMIM 146255), is an autosomal dominant disorder that is caused by heterozygous mutation of the *GATA3* gene [6]. Variable expressivity is common. Parathyroid glands may be absent, hypoplastic, or fibrotic. Levels of parathyroid hormone may be low or unexpectedly normal, and the resulting hypocalcemia may be symptomatic, with different degrees of severity, or asymptomatic. HI manifests early in life, and it is moderate to severe, with a sloping audiogram. Renal involvement is variable, including congenital anomalies of the kidney and urinary tract, and it leads to diverse clinical manifestations. The triad features of HDR syndrome can be occasionally accompanied by defects in other organs, but these are highly variable among affected subjects [7,8].

The *GATA3* gene (OMIM 131320), on 10p14, codes for GATA-binding protein 3 (GATA3), a trans-acting transcription factor that binds the 5′-(A/T)GATA(A/G)-3′ consensus sequence. GATA3 participates in the regulation of the embryonic development of several organs, including the parathyroid glands, the kidney and the urinary tract, and the inner ear, roles that are consistent with the triad of HDR clinical features that result from its altered function. It also regulates the development of many other organs, such as the thymus and mammary glands, roles that may explain the other clinical signs that are occasionally observed in some affected subjects [7,8].

The GATA3 protein (444 residues, 48 kDa) contains two N-terminal transactivation domains and two zinc-finger domains (ZnF), which play different roles. The distal domain (ZnF2, residues 318–342) is essential for binding to the DNA consensus sequence, whereas the proximal domain (ZnF1, residues 264–288) contributes to the stabilization of DNA binding and interacts with other transcription factors. Over 140 pathogenic sequence variants have been reported in *GATA3*, including whole-gene deletions and duplications, nonsense mutations, frameshift and in-frame deletions and insertions, splice-site mutations, and missense mutations [8].

Here, we report a singular case of *GATA3*-related disorder. We identified a *GATA3* missense variant, which affects a residue in ZnF1, and whose pathogenic potential was assessed through functional assays. This variant was found in two brothers, but it was not detected in genomic DNA from blood samples of any of their parents. We could conclude that the variant arose de novo in the father’s germ line. Moreover, the variant does not lead to a complete loss of function of the GATA3 protein, which results in an unexpected phenotype of non-syndromic HI.

## 2. Results

### 2.1. Clinical Characterization

**Figure 1:**
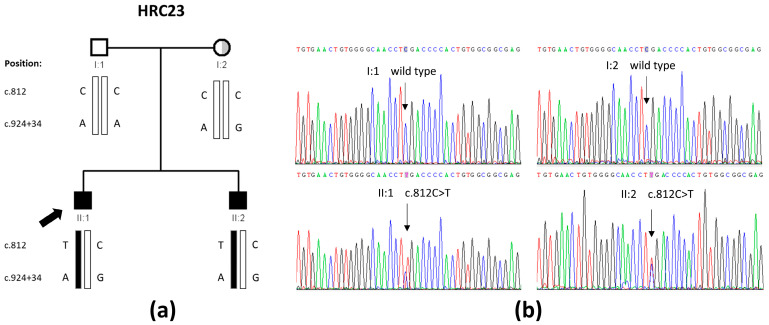
*GATA3* variant that was found in family HRC23 in this study. (**a**) Pedigree showing the segregation of the c.812C>T variant and the c.924+34A>G polymorphism. The half-shaded circle (in grey) represents the unilateral mixed hearing loss of subject I:2. (**b**) Electropherograms from the four family members including the c.812 position within *GATA3*. Both parents are wild type, whereas both brothers are heterozygous for the c.812C>T variant.

**Figure 2:**
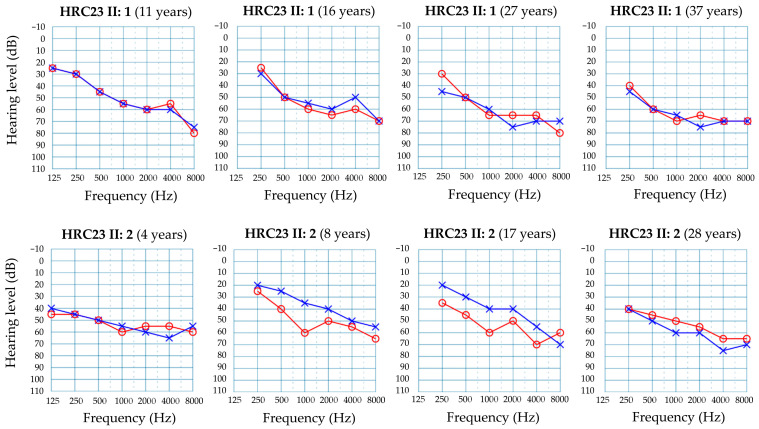
Audiograms from subjects II:1 (**top row**) and II:2 (**bottom row**) at different ages. Only results for air conduction are shown. Red line and circles, right ear. Blue line and crosses, left ear.

Family HRC23 included two affected brothers who were born from non-consanguineous parents (Figure 1a). The two brothers had no other siblings. They shared the same type of hearing impairment, according to their audiological evaluations (Figure 2). The hearing loss is sensorineural, bilateral, symmetrical, and moderate, with slightly sloping audiograms. In the youngest brother (subject II:2, currently 28 years old), it was detected at birth. In the elder brother (subject II:1, currently 37 years old), the hearing loss was diagnosed at age 11 years, a likely delayed diagnosis as his hearing loss is moderate and he was not tested at birth, given that the universal newborn hearing screening program had not been implemented in Spain by then. The hearing losses of both subjects have remained stable to date, as shown by serial audiograms at different ages (Figure 2). No other clinical signs were either reported in their medical records or disclosed upon their clinical examination.

The audiological evaluation of their parents revealed normal hearing in the father (subject I:1) (Figure S1a) and a unilateral severe hearing loss in the mother (subject I:2). She referred multiple ear infections during childhood. Masking audiometry revealed that her hearing loss was mixed (a combination of sensorineural and conductive), with low frequencies being more severely affected (Figure S1b). These audiological features are clearly different from those of her sons and indicated likely different causes, an issue that was taken into account when interpreting the genetic data under different modes of inheritance.

### 2.2. Genetic Study

We performed Whole-Exome Sequencing (WES) on genomic DNA samples from the two affected brothers. Our genetic analysis prioritized candidate variants with a frequency < 0.01 that were shared by both brothers, according to different patterns of inheritance. The two brothers did not share any hemizygous X-linked candidate variant. Prioritizing variants on the hypothesis of an autosomal recessive inheritance, we did not find any homozygous or compound heterozygous candidate variants that were shared by both brothers. Hypothesizing that one of the two expected pathogenic variants might be a structural variant not detected by WES as it occurs sometimes in the so-called monoallelic cases, we prioritized heterozygous variants that were shared by both brothers. This could also detect candidate variants under the hypothesis of an autosomal dominant inheritance. This approach revealed the heterozygous c.812C>T (p.Ser271Leu) variant in exon 4 of the *GATA3* gene (NM_001002295.2) (Figure 1b). This is a non-conservative substitution of a highly conserved residue in ZnF1 (Figure S2).

Sanger sequencing was used to confirm the variant and to investigate its segregation in the family. Unexpectedly, although both brothers were heterozygous for the variant, this was not carried by any of the parents (Figure 1a,b). Genotyping the parents and their two sons for 20 fully informative microsatellite markers excluded a sample shift error and confirmed the parenthood, indicating that the variant had arisen de novo. We noticed that the mother and her two sons, but not the father, carried a heterozygous polymorphism, rs34909898 (c.924+34A>G), in intron 4, very close to the c.812C>T variant, so that both the candidate variant and the polymorphism were contained in the same amplicon. PCR amplification of that DNA segment from the affected brothers was followed by separately cloning and sequencing the two alleles (Figure S3), and it demonstrated that the c.924+34A>G polymorphism was in trans with the c.812C>T variant. Altogether, we concluded that the c.812C>T variant had arisen de novo in the father’s germ line. A germ-line mosaicism would explain the presence of the variant in his two sons. No further investigation of this issue was possible as the father did not give his consent to obtain other biological samples.

Assessment of the pathogenicity of the c.812C>T variant through the ACMG/AMP rules classified it as likely pathogenic (Table 1). Interestingly, this variant was reported in a five-year-old girl with HI but no hypoparathyroidism or any renal disorder at the age of diagnosis [9], which reinforces the conclusion on its involvement in non-syndromic HI.

### 2.3. Clinical Reassessment

The initial diagnosis of non-syndromic hearing loss was reassessed through specific clinical tests (last revision at ages 38 and 27 years old, respectively). Serum levels of calcium, phosphate, and parathyroid hormone were within the normal ranges (Table 2). The patients did not complain of occasional muscle pain, muscle spasms, or paresthesia. Abdominal ultrasonography did not reveal any abnormality in kidneys or urinary tract in any of the two brothers. They had no history of renal infections. Further examination of the patients and their medical records did not reveal any other clinical sign, such as those occasionally observed in subjects with HDR syndrome [10]. Therefore, hearing loss was confirmed to be non-syndromic.

### 2.4. Functional Assays

To further confirm the pathogenicity of the p.Ser271Leu variant, we performed functional assays. COS7 cells (which do not express *GATA3* endogenously) were transiently transfected with pcDNA3 expression vectors containing the whole *GATA3* coding region, either wild type or carrying the p.Ser271Leu variant. Western blot analysis confirmed that GATA3 proteins were correctly synthesized from the wild-type and mutated constructs (Figure S4). We used these constructs to transfect HeLa cells. Immunocytochemistry assays revealed that both the wild-type and mutated GATA3 proteins reached the cell nucleus (Figure S5), indicating that the p.Ser271Leu variant has no effect on the subcellular localization of the protein.

**Figure 3:**
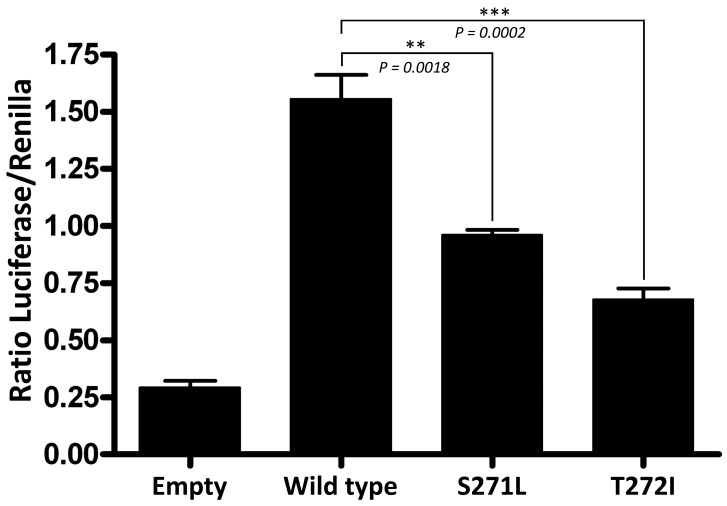
Dual luciferase reporter assay in COS7 cells transiently transfected with the different pcDNA3 plasmid constructs (empty vector, wild-type *GATA3*, and the p.Ser271Leu and p.Thr272Ile mutants) and a plasmid containing the firefly luciferase gene under the control of a *GATA3*-inducible promoter. Constitutively expressed *Renilla* luciferase was used to normalize the firefly luciferase activity. Each experiment was performed by triplicate and all the experiments were performed twice. A two-tailed unpaired Student’s *t* test was used to assess statistical significance.

Finally, a dual-luciferase reporter assay was performed to investigate whether the p.Ser271Leu variant affects the trans-activation capabilities of GATA3. COS7 cells were co-transfected with the different pcDNA3 constructs (wild-type or mutated), a pGL4.23 vector containing the firefly luciferase gene under the control of a GATA3-inducible promoter, and the pRL-null vector containing the constitutively-expressed *Renilla* luciferase gene to normalize the results. Wild-type GATA3 produced a 5.3-fold increase in the expression of the reporter gene, as compared with an empty pcDNA3 vector (Figure 3). The GATA3 protein with the p.Ser271Leu variant produced just a 3.3-fold increase (62% of the wild type). We also assayed a reported pathogenic variant, p.Thr272Ile, which affects the adjacent residue in ZnF1. It produced a 2.3-fold increase in the expression of the reporter (43% of the wild type). The statistical analysis showed that the transactivation activity of the GATA3 protein is significantly reduced by p.Ser271Leu as it occurred with p.Thr272Ile, which supports the classification of p.Ser271Leu as pathogenic (Figure 3).

## 3. Discussion

The clinical and genetic heterogeneity of HI is challenging when trying to reach a genetic diagnosis. A priori determination of the pattern of inheritance in a given pedigree is sometimes misleading. In simplex cases, with no familial history of HI, the hearing loss could have been caused by an environmental factor that remains unidentified. Alternatively, hypothesizing a genetic cause, it was generally thought that most cases may be the result of autosomal recessive inheritance of pathogenic variants. However, de novo variants have a significant contribution (up to 10%), and some of them are heterozygous variants that will be dominantly inherited [11]. In familial cases with two or more affected siblings, finding de novo HI-causing variants is uncommon as they are the result of germ-line mosaicism in one of the parents, as in the case we report here.

Although relatively uncommon, *GATA3* variants rank high among the de novo occurrences in subjects with HI. In a study on a large cohort of subjects with HI, de novo variants were more frequently found in some genes: *MITF* (Melanocyte Inducing Transcription Factor), *GATA3*, *STRC* (stereocilin), and *ACTG1* (actin gamma 1) (in that order) [11]. Other studies searching specifically for mutations in *GATA3* have reported de novo mutations, which account for up to 31% of the cases [8]. Germ-line mosaicism for a de novo *GATA3* pathogenic variant was reported in a pedigree with two affected siblings, like family HRC23, but the parental origin could not be determined [12]. The two siblings had sensorineural hearing loss and hypoparathyroidism, but no renal disease, in contrast to the affected brothers of family HRC23 only having hearing loss.

**Figure 4:**
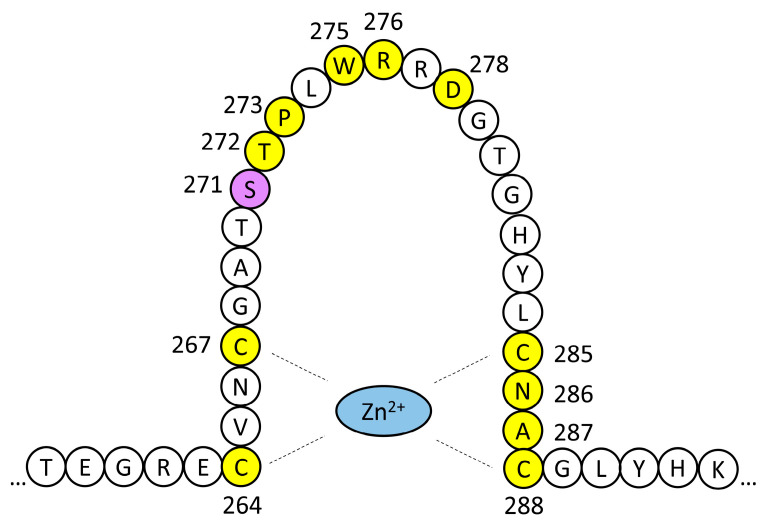
Schematic drawing of GATA3 ZnF1. Residues affected by missense pathogenic variants are highlighted in yellow. The p.Ser271Leu variant of family HRC23 is shaded in purple. The phenotypic effects of these variants are shown in Table S1.

Over 140 germline pathogenic variants of *GATA3* have been reported in cases of HDR syndrome, with variable expressivity [8,10]. About 75% of them are truncating variants (frameshift deletions or insertions, nonsense mutations, splice-site mutations, or whole-gene deletions), and 23% of them are missense variants [8]. Almost without exception, the pathogenetic mechanism seems to be *GATA3* haploinsufficiency. The reported missense mutations cluster in the exons coding for the two zinc-finger domains. Consistently with this location, most of them cause a partial or total abolition of DNA-binding by GATA3, or they result in reduced stability of the DNA–protein complex. The p.Ser271Leu variant we report here affects a residue at the highly conserved sequence of ZnF1 (Figure S2). Pathogenic missense variants affecting 12 out of the 25 residues that constitute ZnF1 have been reported (Figure 4 and Table S1) [9,10,13,14,15,16,17,18,19,20,21,22,23,24,25]. All four cysteine residues that coordinate the zinc ion are affected by pathogenic variants. Another group of variants cluster between residues 271 and 278, including p.Ser271Leu (Figure 4). The relevance of this mutational hotspot is underlined by the fact that some residues are altered by more than one sequence variant (Table S1).

Haploinsufficiency is a common mechanism of disease among genes encoding transcription factors, and it usually results in the variable expressivity of the clinical signs that are characteristic of different syndromic conditions [26]. In fact, only about 54–64% of subjects with *GATA3* pathogenic variants show the complete HDR triad. Sensorineural HI is the most frequent sign (observed in 93–97% of subjects), followed by hypoparathyroidism (87–93%) and renal disorders (61–72%) [7,8]. HI is usually the first feature to be diagnosed [7]. In many cases, other signs are subsequently disclosed by clinical reassessment of the patient. When a diagnosis of non-syndromic HI takes place in childhood, it is difficult to predict whether HI will remain non-syndromic or the other components of the triad may manifest along the patient’s lifespan. In family HRC23, the affected subjects are in their 3rd and 4th decades of life, respectively, and HI has remained non-syndromic to date. It shows the common features of HI in subjects with *GATA3* pathogenic variants [7,8,10]: onset in childhood, moderate severity, non-progression, and sloping audiograms.

Few other subjects with *GATA3* pathogenic variants have been reported to have non-syndromic HI (NSHI). Some of them are subjects from familial cases with variable expressivity: (i) a 29-year-old woman with NSHI, whose father and 32-year-old sister had HI and hypoparathyroidism, all of them carrying the c.856A>G (p.Asn286Asp) variant [13]; (ii) eight subjects with NSHI from one family, which also includes one subject with HI and renal abnormalities, all of them with the p.Phe51LeufsTer144 truncating variant [27]; (iii) two subjects with NSHI from one family, in which three other relatives also had renal disorders, accompanied or not by hypoparathyroidism, all of them carrying the p.Cys288Trp variant [14]. Only two other cases were strictly non-syndromic at the time of the study: all five subjects with NSHI from one family, who carried p.Asp278Asn [15]; and the above mentioned five-year-old girl, with the same mutation that is shared by the two brothers of family HRC23 [9]. Her clinical reassessment did not disclose hypoparathyroidism or any renal disorder. This variant also arose de novo in this girl. The finding of another de novo occurrence of the p.Ser271Leu variant indicates it is a recurring mutation, underlines the relevance of the Ser-271 residue in GATA3 function, and further supports the pathogenicity of the variant and its association with a phenotype of non-syndromic HI.

To date, no direct correlation between the type of mutation and the severity of the HDR syndrome has been established. It has been suggested that missense variants could result in milder phenotypes as they would retain some residual function [8]. In fact, p.Ser271Leu-GATA3 still produced an increase in the expression of the reporter gene in our assays (62% of the wild type). This would be consistent with the non-syndromic HI observed in the three patients with this variant [15] and this work. Moreover, three other cases with non-syndromic HI carried missense variants affecting ZnF1 (p.Asn286Asp, p.Cys288Trp, and p.Asp278Asn). However, phenotypic variability is observed in two of these cases (those carrying p.Asn286Asp, p.Cys288Trp) so that the same mutation results in non-syndromic HI or HDR syndrome. Furthermore, the eight subjects with non-syndromic HI from the family reported in [27] carry a truncating variant. As it has been proposed for other cases of haploinsufficiency for genes encoding transcription factors, genetic modifiers would also play a determinant role in the clinical manifestations and severity of complex syndromes [26]. Of note, GATA3 interacts with its cofactors FOG1 and FOG2 through ZnF1 and cooperates with a variety of other transcriptional factors to regulate gene expression in different tissues. Genetic variants in the genes encoding those GATA3-interacting proteins are candidates to act as those genetic modifiers. Careful follow-up of subjects with only HI to date is needed to determine whether additional clinical signs might manifest with a very delayed onset. Meanwhile, *GATA3* joins other genes encoding transcription factors (*MITF*, *SIX1*, and *SOX10*) whose pathogenic variants predominantly cause syndromic conditions but whose possible contribution to non-syndromic HI should not be ignored [28,29,30].

## 4. Materials and Methods

### 4.1. Human Subjects and Clinical Testing

Family HRC23 was referred to the Department of Genetics of Hospital Universitario Ramón y Cajal for molecular genetic diagnosis. After approval by the Ethical Committee of the Hospital (in accordance with the 1964 Declaration of Helsinki), written informed consent was obtained from all participating subjects. Hearing was evaluated through pure-tone audiometry, with tests for air conduction (frequencies 250–8000 Hz) and bone conduction (frequencies 250–4000 Hz). Masking audiometry was used for unilateral hearing losses. Hearing impairment was classified as mild (21–40 dB HL), moderate (41–70 dB HL), severe (71–95 dB HL), and profound (>95 dB HL), according to the pure-tone average (PTA) threshold levels at 0.5, 1, 2, and 4 kHz. Other clinical data were obtained from the subjects’ medical records and through reexamination (determination of the serum levels of calcium, phosphate and parathyroid hormone, and abdominal ultrasonography) after a genetic diagnosis was established.

### 4.2. DNA Purification, Genotyping, and Sequencing

DNA was extracted from peripheral blood samples by using the Chemagic MSM I automated system (Chemagen, Baesweiler, Germany).

Exome capture was performed using SureSelect Target Enrichment System for 51 Mb (Agilent Technologies, Santa Clara, CA, USA). Paired-end reads of 101-nt were generated on a HiSeq 2000 sequencing platform (Illumina, Inc., San Diego, CA, USA). Reads were aligned against the human reference genome GRCh38/hg38 by using BWA (Burrows-Wheeler Aligner) v0.7.17 [31]. Variant calling was performed using a combination of two different algorithms: VarScanv2.4.6 [32] and GATK v4.0.5.1 [33]. Identified variants were annotated by using Qiagen Clinical Insight Interpret Translational software v9.3.2 (Qiagen, Venlo, The Netherlands) and the Ensembl database v114 [34]. Variants with a minor allele frequency ≥ 0.01 in the genetic databases Exome Aggregation Consortium, 1000-Genomes Project, and dbSNP were excluded from further analysis. Pathogenicity of candidate variants was assessed computationally through multiple predictors as implemented by Qiagen Clinical Insight Interpret Translational software (Qiagen, Venlo, The Netherlands), which was also used to prioritize variants by applying a genetic filter (variants not shared by both siblings were excluded) and a biological filter (variants in genes involved in hearing were selected).

Sanger sequencing was performed to confirm candidate variants and to investigate their segregation within the family. *GATA3* exon 4 and its intronic flanking sequences were PCR-amplified with primers 5′-ATTTTACGTTTCTCCCCCAG-3′ (forward) and 5′- AGAGAGGAAATGAGAAACCCAG-3′ (reverse). To separate the two alleles of each proband, those PCR products were cloned into the pCR2.1 vector by using the TA Cloning Kit (Invitrogen, Waltham, MA, USA) and XL1-Blue competent cells (Agilent Technologies, Santa Clara, CA, USA). Microsatellite markers were amplified using fluorescently labeled primers and PCR conditions as previously reported [35]. Amplified alleles were resolved by capillary electrophoresis in an ABI Prism 3100 Avant Genetic Analyzer (Applied Biosystems, Waltham, MA, USA).

Pathogenicity of the c.812C>T variant was assessed according to the guidelines from the American College of Medical Genetics and Genomics and the Association for Molecular Pathology (ACMG/AMP) [36], as implemented by Varsome v13.3.0 [37]. Scores were subsequently edited manually to take into consideration the expert specification of the ACMG/AMP variant interpretation guidelines for genetic hearing loss [38].

### 4.3. Functional Assays

#### 4.3.1. Plasmid Construction

A full-length *GATA3* cDNA was obtained from the Riken BioResource Center through the National Bio-Resource Project of the MEXT (RIKEN BRC, Tsukuda, Japan) [39,40,41,42]. We inserted it directionally between the *Eco*RI and *Xho*I sites of the pcDNA3 expression vector (Invitrogen, Waltham, MA, USA). Sequence variants c.812C>T (p.Ser271Leu) and c.815C>T (p.Thr272Ile) were introduced by site-directed mutagenesis using Pfu Turbo Polymerase as recommended by the manufacturer (Agilent Technologies, Santa Clara, CA, USA). Design of the mutagenesis probes was carried out by using the QuickChange Primer Design online software v.2.5.3 (Agilent Technologies, Santa Clara, CA, USA). The *GATA3* cDNA sequence of each construct was verified by Sanger sequencing.

#### 4.3.2. Western Blotting

COS7 cells were transiently transfected in 100 mm Petri dishes with 8 µg of pcDNA3 plasmid constructs (wild-type GATA3, p.Ser271Leu-GATA3 or empty vector) in OptiMEM Glutamax medium (Gibco, Waltham, MA, USA) and 30 µL of Lipofectamine 2000 (Invitrogen, Waltham, MA, USA). After incubation during 48h, cells were harvested. Protein extraction and Western blotting were performed using a standard protocol. For Western blot, 2 µL of soluble extract of proteins was loaded in each experiment. We used dilutions of 1/1000 of primary antibody (mouse monoclonal antibody HG3-31, Santa Cruz Biotechnology, Dallas, TX, USA) and 1/10,000 of IRDye 800CW goat anti-mouse IgG secondary antibody (LI-COR Biotechnology, Lincoln, NE, USA). Each assay was performed by triplicate. Image acquisition was performed with the LI-COR Odyssey Platform (LI-COR Biotechnology, Lincoln, NE, USA).

#### 4.3.3. Immunocytochemistry

HeLa cells were transiently transfected in 6-well plates with cover glass using 8 µL of Lipofectamine 2000 (Invitrogen, Waltham, MA, USA) and 4 µg of pcDNA3 plasmid constructs (wild-type GATA3, p.Ser271Leu-GATA3, or empty vector as a negative control to reveal a putative endogenous expression of *GATA3*). Cells were stained using 1/200 anti-GATA3 mouse monoclonal antibody HG3-31 (Santa Cruz Biotechnology, Dallas, TX, USA) and visualized using 1/500 F(ab’)2-Goat anti-mouse Alexa Fluor 488 (Invitrogen, Waltham, MA, USA). Alexa Fluor 546 phalloidin (1/40) (Invitrogen, Waltham, MA, USA) was used to stain the cytoskeleton, and 1/1000 Hoechst 33342 (Invitrogen, Waltham, MA, USA) to stain the nucleus. Each experiment was performed by triplicate.

#### 4.3.4. Luciferase Reporter Assays

We tested the pcDNA3 constructs described in Section 4.3.1. The GATA3-inducible *Il13* promoter was inserted into the pGL4.23 plasmid so that it controlled the expression of the reporter firefly luciferase gene.

About 40,000 COS7 cells were added per well to 24-well plates. Incubation during 24 h resulted in 50–75% confluence, appropriate for an efficient transfection. Cells were transiently transfected with a total of 400 ng of plasmids per well (Table 3), 1.2 µL of FuGENE6 transfection reagent (Promega, Madison, WI, USA), and 100 µL of Opti-MEM glutaMAX medium (Gibco, Waltham, MA, USA). Cells were harvested 48 h after the transfection.

The luciferase assay was performed following the manufacturer’s instructions (Dual-luciferase reporter assay, Promega, Madison, WI, USA), and the luminescence was measured in a FB12 Single Tube Berthold luminometer (Berthold Technologies, Bad Wildbad, Germany). pRL-null (Promega, Madison, WI, USA), which contains *Renilla* luciferase under the control of a minimal promoter for constitutive expression, was used to normalize the firefly (*Photinus pyralis*) luciferase activity. Each experiment was performed by triplicate, and all the experiments were performed twice. A two-tailed unpaired Student’s *t* test was used to assess statistical significance. Graphical representation and statistical analyses were performed using GraphPad Prism 4 software (GraphPad Software, Boston, MA, USA).

## Figures and Tables

**Table 1:** Assessment of pathogenicity of the c.812C>T missense variant.

Gene	Variant		CADD Score	REVEL Score	Minor Allele Frequency	ACMG/AMP Criteria	Classification
	DNA	Protein					
*GATA3*	c.812C>T	p.Ser271Leu	33	0.863	0 (not reported)	PS2 (moderate), PS4 (supporting), PM2 (moderate), PP1 (supporting), PP3 (supporting)	Likely pathogenic

**Table 2:** Serum levels of calcium, phosphate, and parathyroid hormone.

Serum Levels	Subject II:1	Subject II:2	Units	Normal Range
Calcium	10.2	9.8	mg/dL	8.1–10.5
Phosphate	3.2	4.0	mg/dL	2.3–4.7
Parathyroid hormone	23	27	pg/mL	14–72

**Table 3:** Amounts of the different plasmids that were used in each experiment.

Plasmid	Amount			
pGL4.23-*Il13* promoter	200 ng	200 ng	200 ng	200 ng
pcDNA3 empty	100 ng	-	-	-
pcDNA3-*GATA3* wild type	-	100 ng	-	-
pcDNA3-*GATA3* p.S271L	-	-	100 ng	-
pcDNA3-*GATA3* p.T272I	-	-	-	100 ng
pRL-null	100 ng	100 ng	100 ng	100 ng
TOTAL	400 ng	400 ng	400 ng	400 ng

## Data Availability

Data on the pathogenic variant that is reported in this study are available in ClinVar (accession number SCV006080077).

## Supplementary Materials

The following supporting information can be downloaded at https://www.mdpi.com/article/10.3390/ijms26136363/s1.

## Abbreviations

The following abbreviations are used in this manuscript:

ACMG	American College of Medical Genetics and Genomics
AMP	Association for Molecular Pathology
CADD	Combined Annotation Dependent Depletion
HDR	Hypoparathyroidism, Deafness, and Renal Disease
HI	Hearing Impairment
NSHI	Non-Syndromic Hearing Impairment
REVEL	Rare Exome Variant Ensemble Learner
SNP	Single-Nucleotide Polymorphism
WES	Whole-Exome Sequencing
ZnF	Zinc Finger
