# Supplementary material for: De Novo Heterozygous GATA3 Missense Variant Causes an Unexpected Phenotype of Non-Syndromic Hearing Impairment with Apparently Recessive Inheritance
Source: Int J Mol Sci. 2025 Jul 2;26(13):6363. doi: 10.3390/ijms26136363 (PMC12249766; doi:10.3390/ijms26136363)
Supplement: Supplementary file 1 [file ijms-26-06363-s001.zip › ijms-3689381-supplementary.docx]

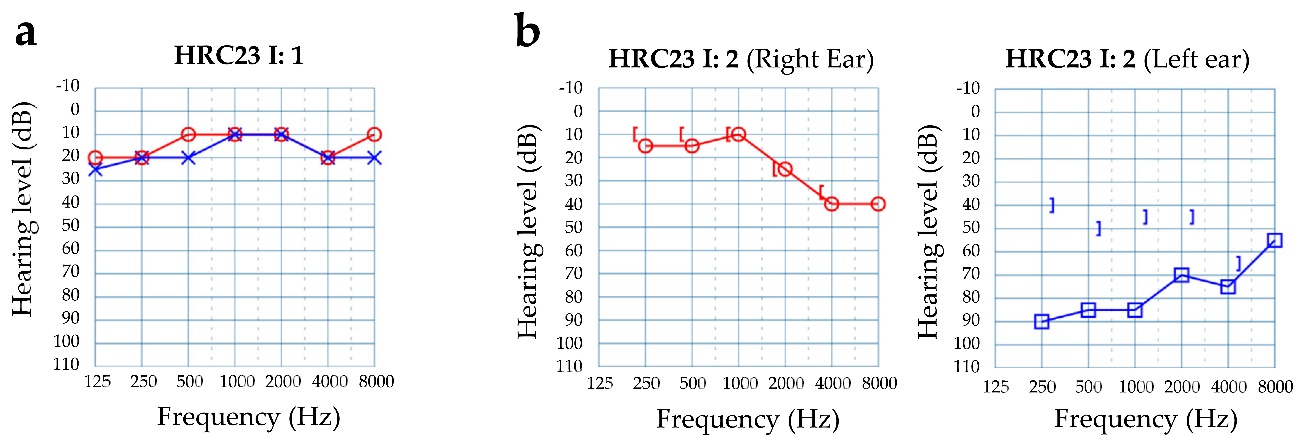


**Figure S1**. Audiograms of the parents of family HRC23. (**a**) Audiogram of subject I:1 (father). Only results for air conduction are shown. Red line and circles, right ear. Blue line and crosses, left ear. (**b**) Masking audiogram of subject I:2 (mother). Circles and squares, air conduction. Brackets, bone conduction. Red symbols, right ear. Blue symbols, left ear.


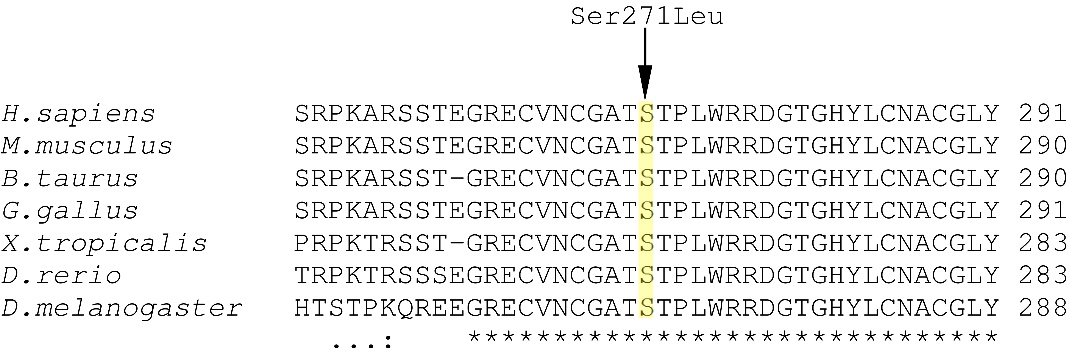


**Figure S2.** Alignment of GATA3 protein orthologous sequences from human and six other animal species. Asterisks indicate identical residues across all sequences; colon, conserved position (residues of strongly similar properties); periods, semi-conserved positions (residues of weakly similar properties). Residues that constitute the ZnF1 are boxed. Sequence accession numbers: *Homo sapiens* (NP_001002295.1); *Mus musculus* (NP_032117.1); *Bos taurus* (NP_001070272.1); *Gallus gallus* (NP_001008444.1); *Xenopus tropicalis* (XP_012814288.1); *Danio rerio* (NP_571286.1); *Drosophila melanogaster* (NP_731211.1).


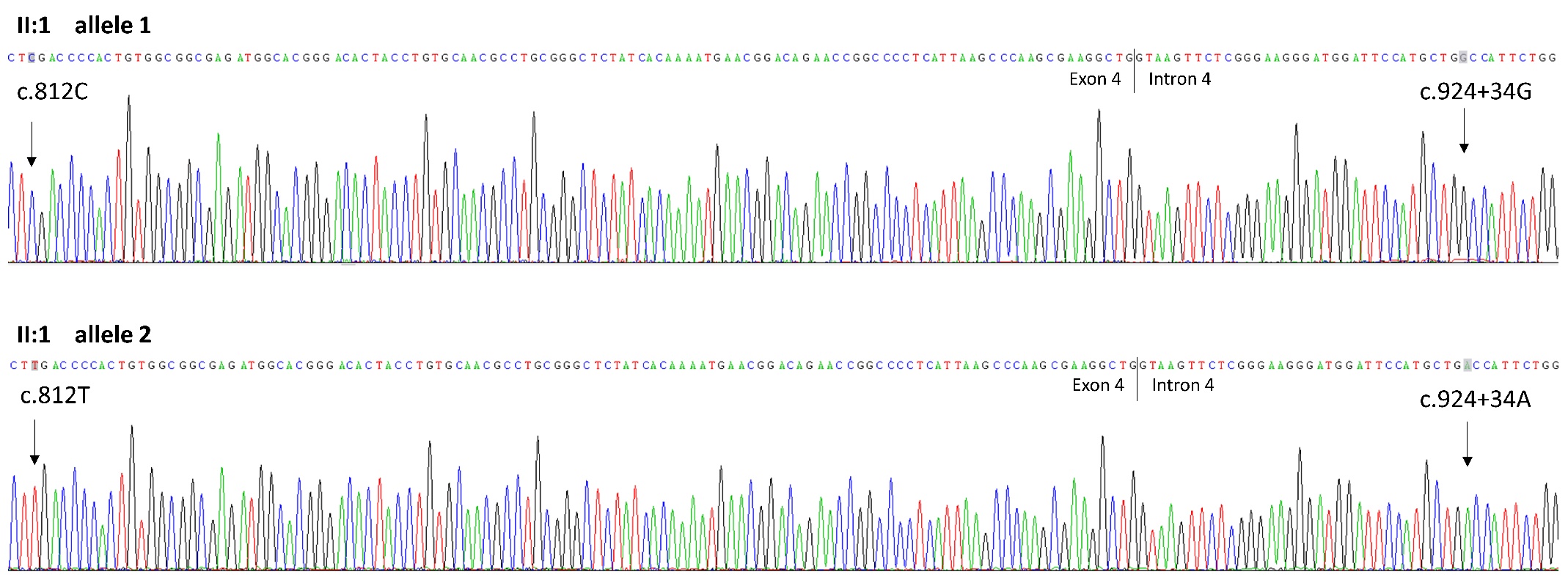


**Figure S3.** Sequences of the two alleles carried by subject II:1 after being cloned separately.


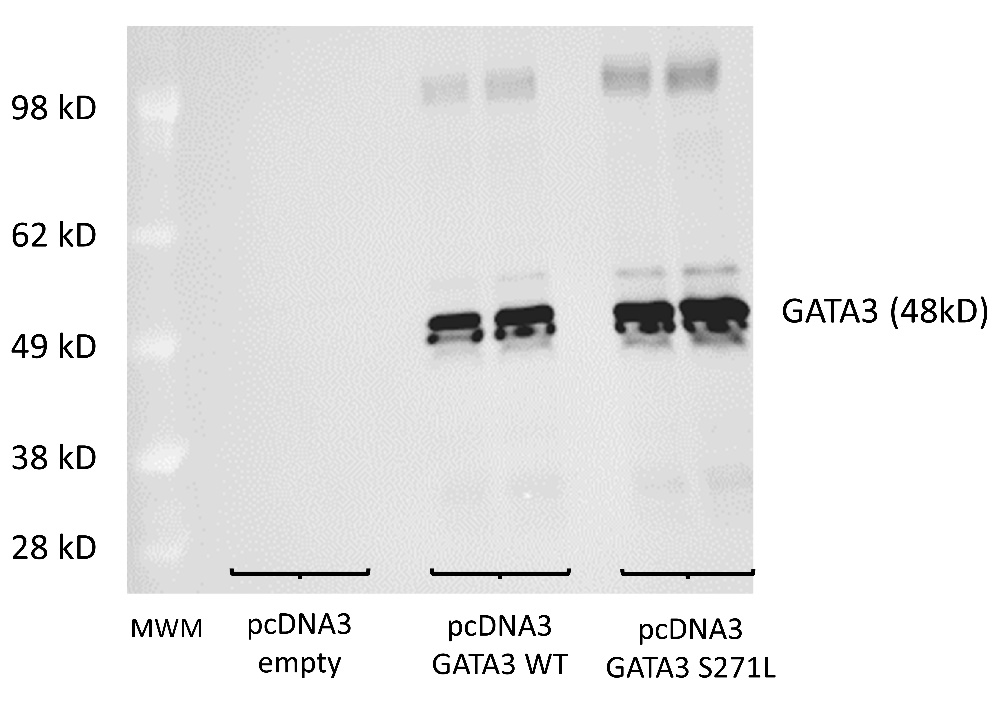


**Figure S4.** Western blot of protein soluble extracts from COS7 cells after transfection with different pcDNA3 constructs. MWM, Molecular Weight Marker.


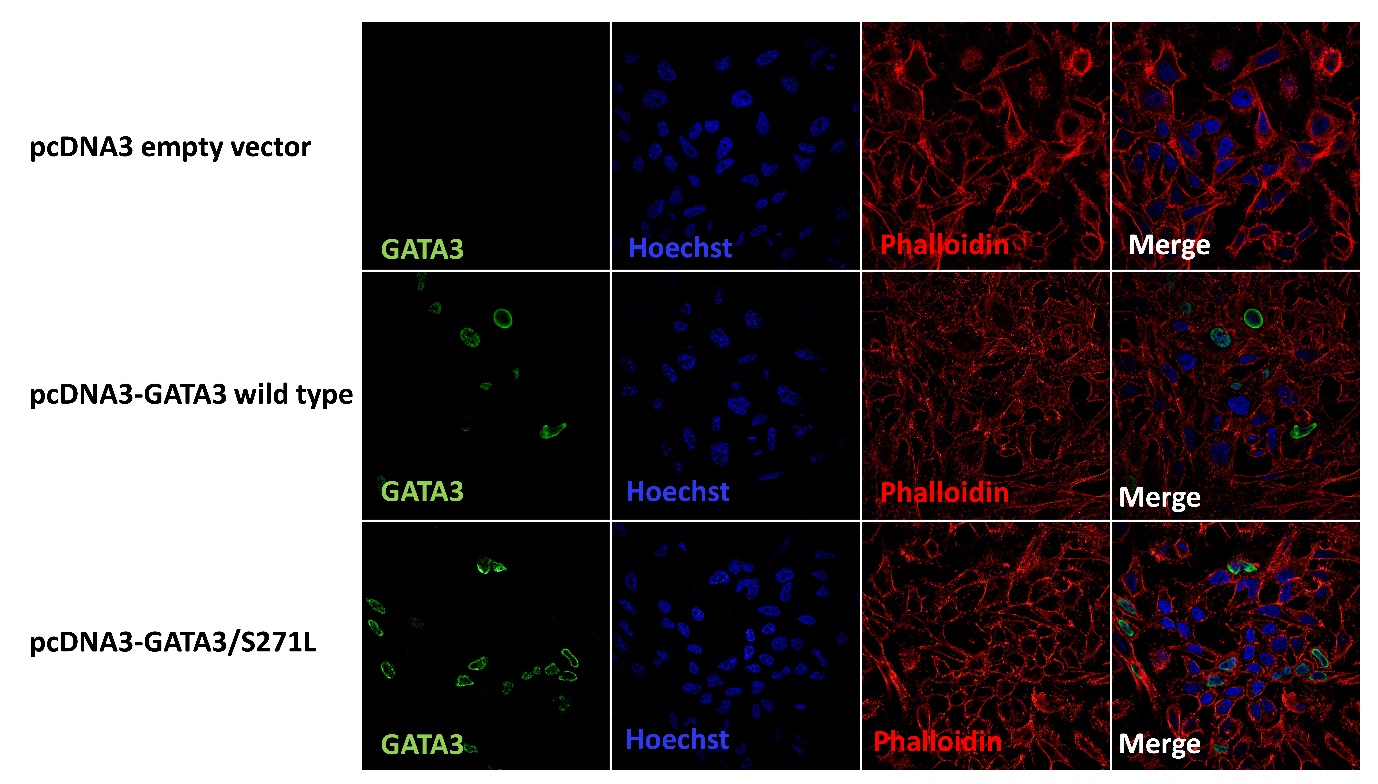


80 µm

**Figure S5.** Fluorescence microscopy images of HeLa cells after transfection with different pcDNA3 constructs, stained with anti-GATA3 antibody HG3-31 plus F(ab')2-goat anti-mouse Alexa Fluor 488 (green), Hoechst 33342 (nuclei; blue), and Alexa Fluor 546-phalloidin (actin filaments; red). Scale bar is the same for all images.

**Table S1**. Pathogenic missense variants affecting the ZnF1 domain of GATA3.

| **Variant** | | **Type of case** | **Inheritance** | **Phenotype (age at diagnosis)** | **Reference** |
| --- | --- | --- | --- | --- | --- |
| **DNA** | **Protein** |  |  |  |  |
| c.791G>A | p.(Cys264Tyr) | Simplex | ND | H: Hypoparathyroidism (40 y)  D: Hearing loss (1 y)  R: Chronic kidney disease and atrophic left kidney (60 y) | [16] |
| c.800G>T | p.(Cys267Phe) | Simplex | *De novo* | H: Hypoparathyroidism (7 mo) | [17] |
| c.812C>T | p.(Ser271Leu) | Simplex  Familial | *De novo*  *De novo* | D: Hearing loss (5 y)  D: Hearing loss (birth, 11 y) | [9]  This work |
| c.815C>T | p.(Thr272Ile) | Simplex | *De novo* | H: Hypoparathyroidism (birth)  D: Hearing loss (8 mo)  R: Pelvic kidney (birth) | [18] |
| c.818C>T | p.(Pro273Leu) | Familial | AD | D: Mild to moderate hearing loss  R: Renal disease | [10] |
| c.823T>A | p.(Trp275Arg) | Familial | AD | H: Hypoparathyroidism (39 y)  D: Hearing loss (childhood)  R:: Left renal hypoplasia (36 y), chronic renal failure | [19] |
| c.824G>T | p.(Trp275Leu) | Simplex | ND | H: Hypoparathyroidism (3 y)  D: Moderate hearing loss (11 y)  R: Renal dysfunction during pregnancy | [20] |
| c.827G>A | p.(Arg276Gln) | Simplex | *De novo* | H: Hypoparathyroidism (13 y)  D: Mild hearing loss (13 y)  R: Pelvic kidney (13 y) | [21] |
| c.827G>C | p.(Arg276Pro) | Familial | AD | H: Hypoparathyroidism  D: Hearing loss (childhood)  R: Right renal agenesis and renal failure (only 1 of 3 subjects) | [22] |
| c.832G>A | p.(Asp278Asn) | Familial | AD | D: Severe to profound hearing loss | [15] |
| c.855C>G | p.(Cys285Trp) | Simplex | *De novo* | H: Hypoparathyroidism  D and R: not specified (data from five cases were reported in aggregate) | [23] |
| c.856A>G | p.(Asn286Asp) | Familial | AD | H: Hypoparathyroidism (2 subjects)  D: Hearing loss (3 subjects) | [13] |
| c.860C>A | p.(Ala287Asp) | Simplex | ND | H: Hypoparathyroidism  D: Hearing loss (childhood)  R: Hypoplastic right kidney and chronic kidney disease | [24] |
| c.862T>C | p.(Cys288Arg) | NR | NR | H: Hypoparathyroidism  D: Hearing loss  R: Renal disease | [10] |
| c.863G>A | p.(Cys288Tyr) | Familial | AD | H: Hypoparathyroidism  D: Moderate hearing loss  R: Right renal agenesis | [25] |
| c.864C>G | p.(Cys288Trp) | Familial | AD | H: Hypoparathyroidism (1 of 5 subjects)  D: Hearing loss (all 5 subjects)  R: Glomerulonephritis (3 subjects) | [14] |

Variants have been named using the NM_001002295.2 reference *GATA3* cDNA sequence. AD, autosomal dominant; ND, not determined; NR, not reported; y, years; mo, months.
